# Aflatoxin Exposure during Early Life Is Associated with Differential DNA Methylation in Two-Year-Old Gambian Children

**DOI:** 10.3390/ijms22168967

**Published:** 2021-08-20

**Authors:** Akram Ghantous, Alexei Novoloaca, Liacine Bouaoun, Cyrille Cuenin, Marie-Pierre Cros, Ya Xu, Hector Hernandez-Vargas, Momodou K. Darboe, Andrew M. Prentice, Sophie E. Moore, Yun Yun Gong, Zdenko Herceg, Michael N. Routledge

**Affiliations:** 1 International Agency for Research on Cancer, 150, Cours Albert Thomas, 69372 Lyon, France; GhantousA@iarc.fr (A.G.); anovoloaca@yahoo.fr (A.N.); BouaounL@iarc.fr (L.B.); CueninC@iarc.fr (C.C.); CrosMP@iarc.fr (M.-P.C.); Hector.HERNANDEZ-VARGAS@lyon.unicancer.fr (H.H.-V.); HercegZ@iarc.fr (Z.H.); 2 School of Medicine, University of Leeds, Leeds LS2 9JT, UK; xuya5@sysu.edu.cn; 3 Guangdong Provincial Key Laboratory of Malignant Tumor Epigenetics and Gene Regulation, Medical Research Center, Sun-Yat Sen University, Guangzhou 510006, China; 4 Cancer Research Centre of Lyon (CRCL), Université de Lyon, 69008 Lyon, France; 5 MRC Unit the Gambia at the London School of Hygiene and Tropical Medicine, Atlantic Boulevard, Fajara, Banjul P.O. Box 273, The Gambia; mdarboe@mrc.gm (M.K.D.); Andrew.Prentice@lshtm.ac.uk (A.M.P.); sophie.moore@kcl.ac.uk (S.E.M.); 6 Department of Women and Children’s Health, King’s College London, St Thomas’ Hospital, London SE1 7EH, UK; 7 School of Food Science and Nutrition, University of Leeds, Leeds LS2 9JT, UK; y.gong@leeds.ac.uk; 8 School of Food and Biological Engineering, Jiangsu University, Zhenjiang 212013, China

**Keywords:** aflatoxin B1, aflatoxin albumin adducts, DNA methylation, Gambian children

## Abstract

*Background*: DNA methylation is an epigenetic control mechanism that may be altered by environmental exposures. We have previously reported that in utero exposure to the mycotoxin and liver carcinogen aflatoxin B1 from the maternal diet, as measured using biomarkers in the mothers’ blood, was associated with differential DNA methylation in white blood cells of 6-month-old infants from The Gambia. *Methods*: Here we examined aflatoxin B1-associated differential DNA methylation in white blood cells of 24-month-old children from the same population (*n* = 244), in relation to the child’s dietary exposure assessed using aflatoxin albumin biomarkers in blood samples collected at 6, 12 and 18 months of age. HM450 BeadChip arrays were used to assess DNA methylation, with data compared to aflatoxin albumin adduct levels using two approaches; a continuous model comparing aflatoxin adducts measured in samples collected at 18 months to DNA methylation at 24 months, and a categorical time-dose model that took into account aflatoxin adduct levels at 6, 12 and 18 months, for comparison to DNA methylation at 24 months. *Results*: Geometric mean (95% confidence intervals) for aflatoxin albumin levels were 3.78 (3.29, 4.34) at 6 months, 25.1 (21.67, 29.13) at 12 months and 49.48 (43.34, 56.49) at 18 months of age. A number of differentially methylated CpG positions and regions were associated with aflatoxin exposure, some of which affected gene expression. Pathway analysis highlighted effects on genes involved with with inflammatory, signalling and growth pathways. *Conclusions*: This study provides further evidence that exposure to aflatoxin in early childhood may impact on DNA methylation.

## 1. Introduction

Epigenetic alterations such as DNA methylation changes have been proposed as important mediators of long-term health effects of environmental exposures [1,2]. DNA methylation patterns are set during early development, where they participate in the establishment and maintenance of cell lineages, but these patterns may be modified during life [3]. Consistent with the role of DNA methylation as the key mechanism for control of gene expression [4], changes in DNA methylation patterns have been observed with aberrant transcriptional changes in cancer and other diseases [5,6,7,8]. There is increasing evidence that environmental exposures, including cigarette smoking, heavy metals and persistent organic pollutants can alter DNA methylation in humans [1,2]. Changes in DNA methylation during foetal development have been associated with adverse birth outcomes, with evidence that these changes can also influence disease in later life [9,10], although the precise underlying mechanism remains poorly understood.

Aflatoxin B1 (AFB1) is a mycotoxin that is highly toxic and carcinogenic [11]. The *Aspergillus* species of fungi that produce AFB1 commonly grow on food crops, including staples such as maize and peanuts, in warm humid climates, leading to frequent contamination of these crops. At particular high risk of exposure to AFB1 are subsistence farmers in sub-Saharan Africa, where climate conditions, poor storage practices and low socioeconomic status combine to increase intake of contaminated foods. Numerous studies using biomarkers of exposure have shown chronic and occasionally very high levels of AFB1 exposure among rural populations in sub-Saharan Africa [12,13,14,15,16]. Importantly, many studies have linked this exposure to adverse health outcomes, including liver cancer, acute liver disease, and child growth impairment [17,18]. AFB1 is known to induce mutagenic DNA adducts that are likely to be involved in the development and progression of cancer [19]. The mechanism for other health effects have not been determined, but we have previously shown that in utero exposure to aflatoxin, as assessed by levels of aflatoxin-albumin adducts (AF-alb) in serum of the mothers, was associated with differential DNA methylation in a number of genes in the white blood cells (WBC) of the infants [20].

In this study, we examined the impact of early-life exposure to aflatoxin on the DNA methylome patterns, using a new-generation bead-chip arrays with genome coverage. We established the methylome profiles of young children’s DNA from Gambian children (who had been naturally exposed to aflatoxin in their diet, as assessed by measuring AF-alb in serum) at two years of age. Further analyses revealed that exposure to aflatoxin during the first two years of life is associated with differential DNA methylation in a range of genes and pathways, some of which directly relate to known health effects of aflatoxin exposure.

## 2. Results

### 2.1. Aflatoxin Exposure over Time in Early Life

**Figure 1:**
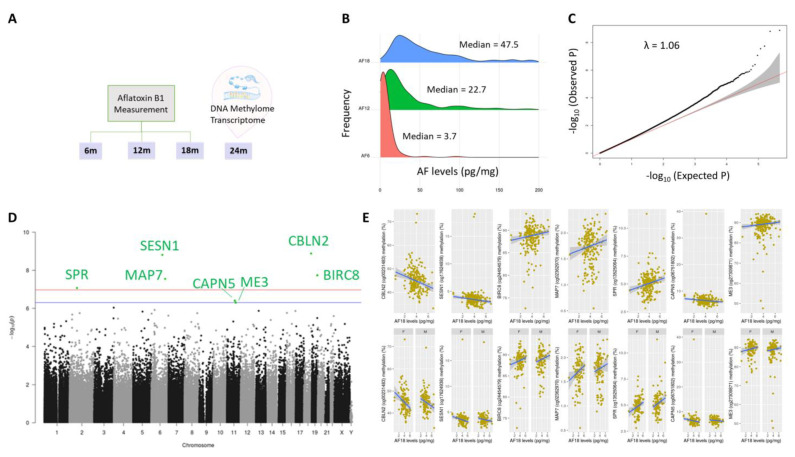
(**A**) Study design of the project. Aflatoxin-albumin adducts were measured in the infants at 6, 12 and 18 months old. The DNA methylome and transcriptome were profiled in infants’ blood, collected at 24 months. (**B**) Aflatoxin B1 levels at 6, 12 and 18 months during infancy. (**C**) Q-Q plot and the genomic inflation factor (lambda) of the DMP analysis, based on the adjusted model using AF18. (**D**) Manhattan plot showing the significant DMPs, being above the Bonferroni (red) and FDR (blue) lines. (**E**) Scatter plots of methylation vs. AF18 levels for the DMPs, stratified in the second row by child sex.

Characteristics of the children and geometric mean values for AF-alb are given in Table 1. We observed an increase in median values of AF-alb with time (3.7 pg/mg albumin at 6 months, 22.7 pg/mg albumin at 12 months and 47.5 pg/mg albumin at 18 months; Figure 1B), although an increase between 6 and 12 months was not observed in all individuals (Supplementary file S1B).

### 2.2. DNA Methylation Markers of Aflatoxin Exposure and Associated Biological Pathways: Continuous Model

In the continuous model, the Q-Q plot (Figure 1C) shows limited inflation (lambda close to 1.0), giving a low risk of false positives. Seven differentially methylated positions (DMPs) had a difference in DNA methylation associated with aflatoxin exposure, with an FDR-adjusted *p*-value < 0.5 (Figure 1D; Supplementary file S2). The changes in DNA methylation according to AF-alb continuous values for these DMPs are shown in Figure 1E, which also shows that there was no difference according to sex of the children.

**Figure 2:**
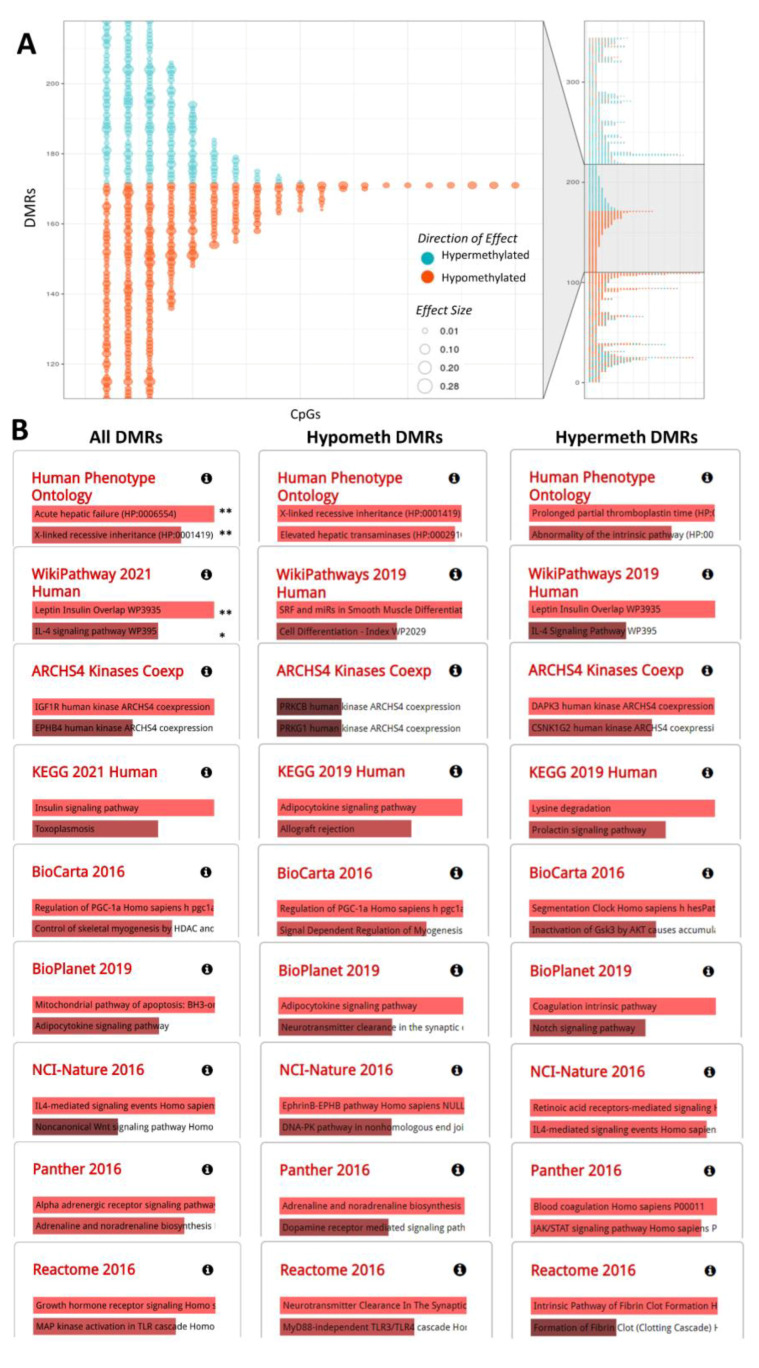
(**A**) DMRs obtained from the AF18 continuous model are represented on the *y*-axis and their constituent CpGs on the *x*-axis. Hyper- and hypo-methylated CpGs in relation to AF levels are shown in blue and orange circles, respectively, and the size of the circle is proportional to the effect size (being the regression coefficient). The middle zoomed cluster represents DMRs encompassing three or more CpGs showing a 100% consistent direction of effect. (**B**) Ontology (first row) and pathway (other rows) analysis using EnrichR was performed on all DMRs (first column) and then stratified by hyper- and hypo-methylated DMRs (second and third columns, respectively). Hyper- and hypo-DMRs were defined as those in which at least 2/3 of the CpGs show a consistent direction of effect towards hyper- and hypo-methylation, respectively. Results are ranked by *p*-value (the most significant being on top), and only the two most significant pathways (*p* < 0.05) are shown for each database. ** FDR < 0.05; * FDR < 0.1. More results for Human Phenotype Ontology of All DMRs are shown in Table S3A as more than two significant (FDR < 0.1) results were obtained. In ontology analysis, only Human Phenotype Ontology is reported here as it yielded the most significant results; GO Biological Process did not yield significant ontologies (FDR < 0.1). A detailed report for the ontology and pathway analyses is available through the following links: All DMRs: https://amp.pharm.mssm.edu/Enrichr/enrich?dataset=b4e3a404c15ef001a7c30c1a13e1d249#; Hypometh DMRs: https://amp.pharm.mssm.edu/Enrichr/enrich?dataset=9357fa44b29ebb8dc326803ebf26925e; and Hypermeth DMRs: https://amp.pharm.mssm.edu/Enrichr/enrich?dataset=fcbe92f8726688e4eee8961de6e89d03 (Accessed on 23 March 2020).

In addition to these DMPs, there were 509 DMRs showing differences in relation to continuous AF-alb levels at 18 months (Figure 2A; Supplementary file S3), with a large proportion of these encompassing at least three CpG sites with a consistent direction of effect. Ontology analysis of the results (Figure 2B) showed that the DMRs are enriched in genes associated with acute hepatic failure, as well as others associated with developmental disorders including intrauterine growth retardation (Supplementary file S4; e.g., micropenis, short neck and intrauterine growth retardation). In terms of biological mechanisms, DNA methylation markers associated with aflatoxin exposure were enriched in inflammatory pathways (particularly the hypermethylated DMRs; e.g., IL4 signalling), nervous system signalling (particularly the hypomethylated DMRs; e.g., alpha adrenergic receptor signalling, EphrinB-EPHB pathway, adrenaline and noradrenaline biosynthesis, and neurotransmitter clearance) and signalling events affecting the growth hormone-insulin-obesity axis (enriched in both hyper- and hypo-methylated DMRs; e.g., leptin insulin overlap, IGF1R, insulin signalling, regulation of PGC-1a, growth hormone receptor signalling, and adipocytokine signaling; Figure 2B).

### 2.3. DNA Methylation Markers of Aflatoxin Exposure and Associated Biological Pathways: Categorical Time-Dose Model

**Figure 3:**
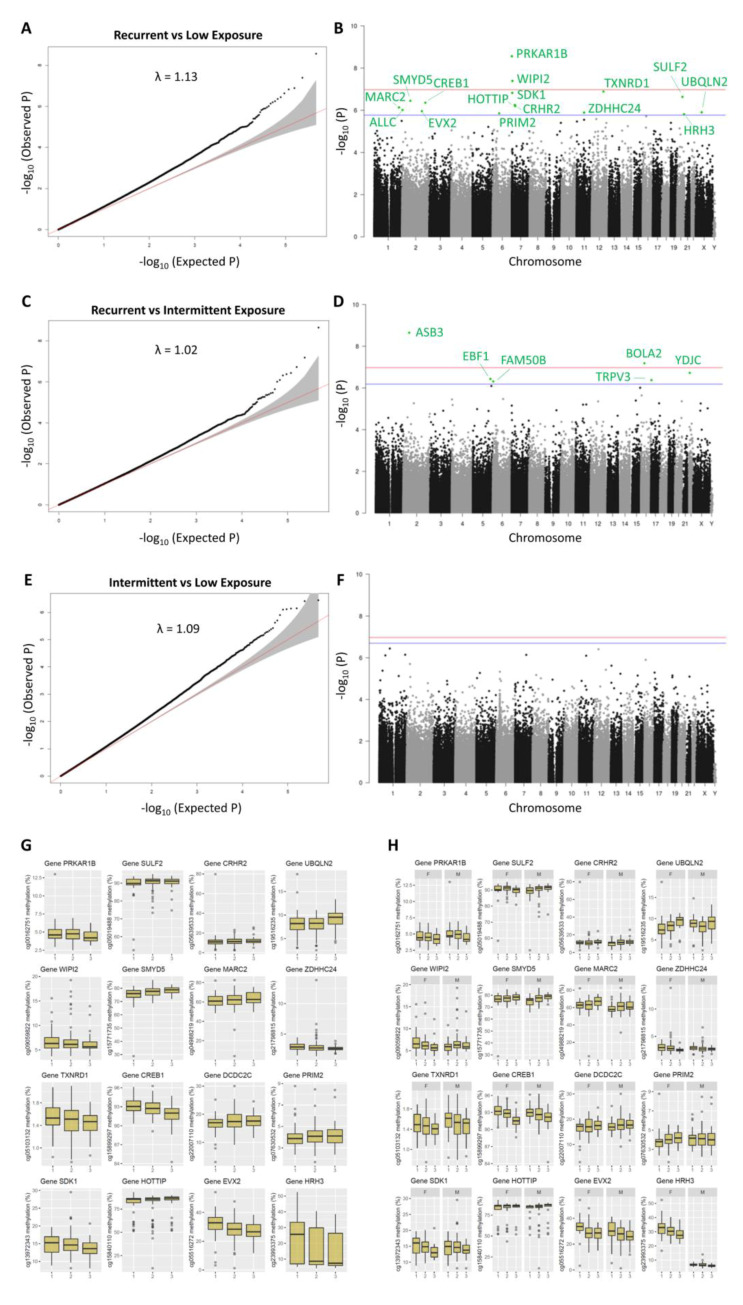
Categorical time-dose DMP analysis (recurrent, intermittent or low exposure) of aflatoxin levels over the period of 6, 12 and 18 months postnatally. (**A**,**C**,**E**) Q-Q plots and the genomic inflation factor (lambda) of the DMP analyses. (**B**,**D**,**F**) Manhattan plots showing the significant DMPs, being above the Bonferroni (red) and FDR (blue) lines. Results for the recurrent vs. low, recurrent vs. intermittent, and intermittent vs. low aflatoxin exposure models are shown in (**A**–**F**), respectively. (**G**) Scatter plots of the significant DMPs (FDR < 0.05) obtained from the recurrent vs. low exposure model, highlighting the methylation levels in relation to recurrent (1), intermittent (2) and low (3) exposures and stratified in (**H**) by child sex.

In the categorical analysis, the Q-Q plots also show low inflation, with lambda values being close to 1.0 (Figure 3A,C,E). The largest number of hits was seen for the recurrent vs. low exposure model, followed by the recurrent vs. intermittent and intermittent vs. low models (Figure 3B,D,F), suggesting a time-dose response. The DMPs (FDR < 0.05) of the recurrent vs. low (Figure 3B) and recurrent vs. intermittent (Figure 3D) exposure models are further detailed in Supplementary files 5 and 6, respectively. No DMPs (FDR < 0.05) were obtained in the intermittent vs. low exposure model (Figure 3F). The methylation levels for each category (low, recurrent and high) are shown in Figure 3G, visualizing the effect of the time-dose response of exposure on methylation. Some sex-specific effects are seen for DMPs: in *UBQLN2*, *WIPI2*, *PRIM2* and *HRH3*, the effect was only seen in girls, and in *SULF2*, boys showed an inverse (though minor) direction of effect relative to girls.

**Figure 4:**
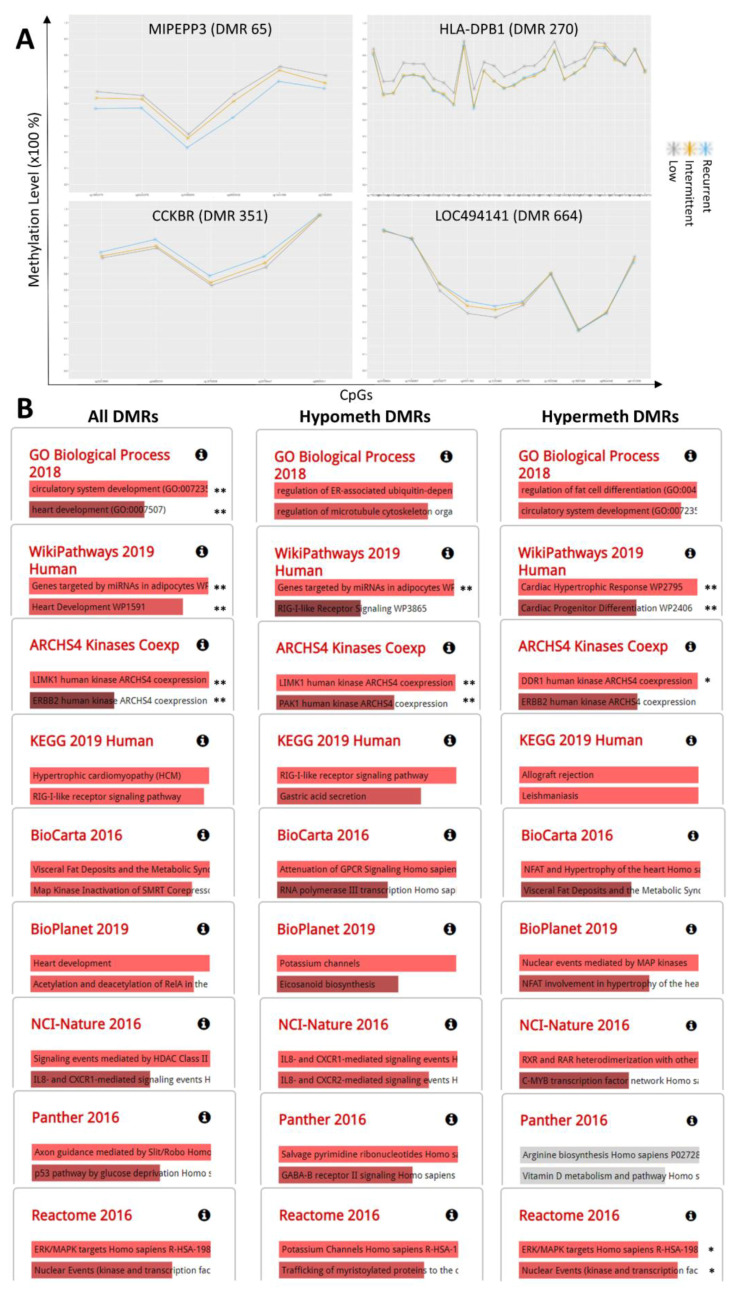
(**A**) Top DMRs obtained from the AF recurrent vs. low AF exposure adjusted model, with their methylation levels on the *y*-axis and their constituent CpGs on the *x*-axis. The DMRs in the MIPEPP3 and HLA-DPB1 genes represent the two DMRs with the largest number of CpGs, each having an effect size ≥ 5%. Among the remaining DMRs that pass this criterion, those in the CCKBR and LOC494141 genes are shown because they encompass at least three consecutive CpGs with an effect size ≥ 5%. The detailed list of DMRs from this model are shown in Tables S5A.1 and S5A.2, the latter focusing on the DMRs harbouring at least one CpG with an effect size ≥ 5%. The DMRs of the recurrent vs. intermittent and the intermittent vs. low exposure models are shown in Tables S5B and S5C, respectively. (**B**) Ontology (first row) and pathway (other rows) analysis using EnrichR was performed on all DMRs (first column) and then stratified by hyper- and hypo-methylated DMRs (second and third columns, respectively) of the recurrent vs. low exposure model. Hyper- and hypo-DMRs were defined as those in which at least 2/3 of the CpGs show a consistent direction of effect towards hyper- and hypo-methylation, respectively. Results are ranked by *p*-value (the most significant being on top), and only the two most significant pathways (*p* < 0.05) are shown for each database. ** FDR < 0.05; * FDR < 0.1. More results for GO Biological Process of All DMRs, ARCHS4 Kinases Coexp of All DMRs and ARCHS4 Kinases Coexp of Hypomethylated DMRs are shown in Tables S3B, S3C and S3D, respectively, as more than two significant (FDR < 0.1) results were obtained. In ontology analysis, only GO Biological Process is reported here as it yielded the most significant results; Human Phenotype Ontology did not yield significant ontologies (FDR < 0.1). A detailed report for the ontology and pathway analyses is available through the following links: All DMRs https://amp.pharm.mssm.edu/Enrichr/enrich?dataset=4b3cff0bf36747174475beaa6d748b18#; Hypometh DMRs: https://amp.pharm.mssm.edu/Enrichr/enrich?dataset=55785d6f4752f5e4938d94428fc3779e#; and Hypermeth DMRs: https://amp.pharm.mssm.edu/Enrichr/enrich?dataset=c58eb28eecd53247b5cc2b973eb21009# (Accessed on 4 September 2020).

There were 714 DMRs obtained in the Recurrent vs. Low AF-alb model (Figure 4; see also Supplementary file S7). 51 of these encompass at least one CpG with an effect size ≥ 5% (Supplementary file S8). The top four DMRs are shown here (Figure 4A). The DMRs in the *MIPEPP3* and *HLA-DPB1* genes represent the two DMRs with the largest number of CpGs, each having an effect size ≥ 5%. Among the remaining DMRs that pass this criterion, those in the *CCKBR* and *LOC494141* genes are shown because they encompass at least three consecutive CpGs with an effect size ≥ 5%. Except for *HLA-DPB1*, the other three DMRs show a time-dose response, with the effect sizes of intermittent exposure being in between those of the recurrent and low exposures. For *HLA-DPB1*, intermittent and recurrent exposures had almost identical methylation levels highlighting a potentially irreversible hypomethylation effect of AF on this gene, relative to low exposure. Other DMRs with less stringent criteria are shown in Supplementary file S9 (DMRs encompassing at least two consecutive CpG with effect size ≥ 5%, a max of one CpG being non-unimodally distributed, and 100% consistent direction of effect among its CpGs that have an effect size ≥ 3%). The 714 DMRs, particularly the hypermethylated regions, were enriched in genes associated with circulatory and heart development as well as cardiac-related pathways (Figure 4B; see also Supplementary file S10; e.g., circulatory system development, heart development, hypertrophic cardiomyopathy, cardiac hypertrophic response, and cardiac progenitor differentiation). Similar to the pathways enriched in DMRs from the continuous AF-alb model, the DMRs for the recurrent vs. low model were also enriched in nervous system- (particularly the hypomethylated DMRs; e.g., axon guidance and GABA-R receptor II signalling), inflammatory- (particularly the hypomethylated DMRs; e.g., IL8- and CXCR1-mediated signalling) and adipose- (enriched in both hyper- and hypo-methylated DMRs; e.g., genes targeted by miRNAs in adipocytes, visceral fat deposits and metabolic syndrome, eicosanoid biosynthesis, and regulation of fat cell differentiation) related pathways. The most enriched ARCHS4 kinase pathway, LIMK1, associates with both nervous system (stimulate axon outgrowth) and cardiac- (predisposing to Supravalvular Aortic Stenosis) related pathways (GeneCards) (see also Supplementary file S11).

### 2.4. Genomic Distribution and Functional Characterization of Identified DNA Methylation Markers of Aflatoxin Exposure

**Figure 5:**
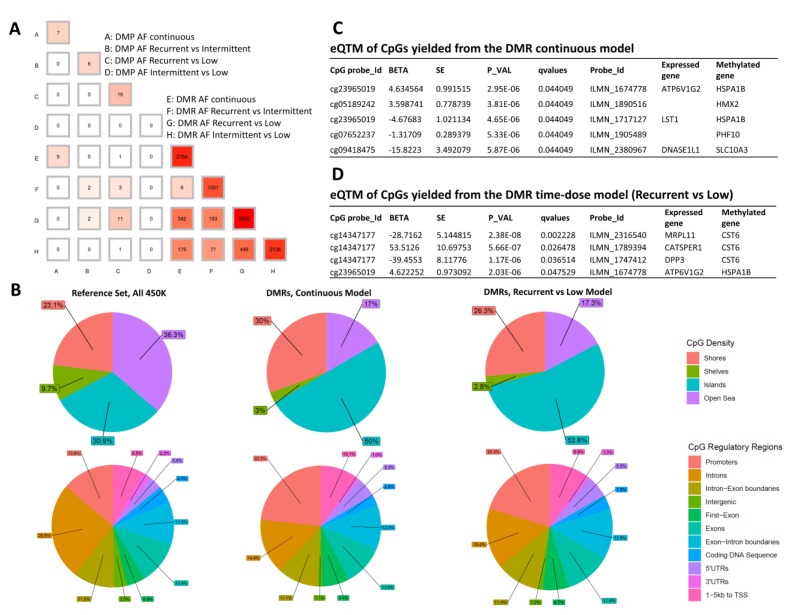
(**A**) The number of CpGs overlapping between the different reported models, A–H. (**B**) CpG Density (upper row) and Regulatory Region (lower row) distributions of the DMRs relative to the whole 450 K probe set (used as a reference). (**C**,**D**) expression Quantitative Trait Methylation (eQTM) analysis showing significant association (FDR < 0.05) between CpG methylation and RNA expression of nearby genes (within ± 250 Kb). The CpGs yielded from the DMR continuous and Recurrent vs. Low exposure models were used in (**C**,**D**), respectively. In both models, the eQTMs were adjusted for AF levels in order to eliminate any potential direct effect of AF on expression.

Figure 5A shows the overlap in hits between the different reported models. Five out of seven CpGs are common between the DMP and DMR analyses of the AF-alb continuous model (A vs. E). Eleven out of 16 CpGs are common between the DMP and DMR analyses of the AF-alb Recurrent vs. Low categorical model (C vs. G). These examples show that the DMP and DMR analyses are quite consistent in the two major models. DMRs from the continuous or categorical time-dose models are similarly enriched in CpG islands and shores and depleted in shelves and open sear regions relative to the HM450 reference set (Figure 5B). DMRs from either model are also similarly enriched in promoter, 1–5 Kb to TSS, 5′UTR and first exon regions and depleted from exons, introns and intergenic regions relative to the 450K reference set. The DNA methylation at certain CpG loci affects RNA expression of nearby genes (FDR < 0.05), with the direction of effect being positive for some eQTMs and negative for others (Figure 5C,D). *ATP6V1G2* expression was associated with differential methylation of cg23965019 (*HSPA1B* gene) in both the continuous (Figure 5C) and categorical time-dose (Figure 5D) models.

## 3. Methods

### 3.1. Study Population and Blood Sampling

The current work was part of a study on the impact of aflatoxin and child growth, which was a sub-study embedded within the Early Nutrition and Immune Development (ENID) trial (ISRCTN49285450). Pregnant mothers were recruited into the ENID trial from the rural, West Kiang region of The Gambia. Full details of the ENID trial protocol [21] and aflatoxin and growth sub-study [18] have been described elsewhere.

Samples used in the current DNA methylation study were from 243 children born into ENID between May 2011 and December 2012. Children were followed up over two years, with blood samples taken at 6, 12, 18 and 24 months of age. For the purpose of the current analysis we used serum extracted from samples at 6, 12 and 18 months and WBC extracted from samples taken at 24 months.

### 3.2. AF-alb Analysis

The analysis of AF-alb followed the method first described by Chapot and Wild [22]. Albumin was extracted from serum samples, hydrolysed using pronase and purified with Sep-pak C-18 cartridges (Waters, Milford, MA, USA). AF-alb was quantified using a competitive ELISA [22,23] using a rabbit anti aflatoxin-Cl2-BSA polyclonal antibody (a gift from Christopher Wild, IARC) as the primary antibody. The detection limit (LOD) was 3 pg AF-lysine equivalents per mg albumin, and each ELISA included three positive and one negative control samples for quality control. All samples were measured in duplicate for each ELISA batch and repeated at least two times on separate days to confirm the results. Results were accepted when values within each ELISA had a %CV below 10% and samples tested on separate occasions had a %CV below 15%. If samples lay above the linear part of the standard curve, the ELISA was repeated at a suitable dilution. This ELISA has been validated for measuring aflatoxin albumin biomarker levels against dietary aflatoxin intake, and also against an LC-MS for measuring aflatoxin lysine. Whilst the ELISA consistently gives Af-alb values that are approximately three-fold higher than aflatoxin lysine values measured by LC-MS the results between methods correlate for the same samples [24]. The AF-alb values are, therefore, suitable for use in analyses to compare variations in aflatoxin exposure in relation to health outcomes or other variables such as differential DNA methylation.

### 3.3. Bisulfite Conversion

Bisulfite conversion was performed using the EZ-96 DNA Methylation kit (Zymo Research Corporation, Irvine, CA, USA), and its efficiency was tested using PCR primers that were specific either to bisulfite-converted or to non-modified DNA and spanning the *NBR2* region.

### 3.4. Genome-Wide Methylation Array

Sample preparation and hybridization to the HM450 BeadChip arrays (Illumina, San Diego, CA, USA), which covers ~450,000 CpG sites, were performed at the International Agency for Research on Cancer (IARC). From the 600 ng of bisulfite-converted DNA per sample, 200 ng was used for hybridization on the array. Each array consisted of 96 samples distributed equally among 8 chips in a completely randomized manner so that batch effects (e.g., sample position and intra and inter-variability in arrays and chips) do not completely confound with biological covariates of interest. The analysis on the bead array was conducted following the recommended protocols for amplification, labelling, hybridization and scanning [25].

### 3.5. DNA Methylome Data Pre-Processing

Data pre-processing and analysis were performed using R/Bioconductor packages (http://www.bioconductor.org/, accessed on 27 December 2019). Raw data of the samples constituted of 485,512 CpG probes from the HM450 array and were loaded from IDAT files and handled in the minfi package [26] to calculate the methylation level at each CpG as the beta-value (β = intensity of the methylated allele (M)/(intensity of the unmethylated allele (U) + intensity of the methylated allele (M) + 100)). Data quality was assessed using box plots for the distribution of methylated and unmethylated signals, unsupervised clustering showing close agreement between technical replicates, array-wide multi-dimensional scaling plots and unsupervised clustering to identify sample outliers, and multidimensional scaling plots clustering samples into two groups representing infant sex. Filtering of cross-reactive probes (*n* = 14,549) [27] and low quality probes (*n* = 1183) (more than 5% missing values per probe) resulted in a total of 469,853 CpGs to analyze. Beta values were normalized with Functional normalization (Funnorm, minfi R package) that was shown to perform equally good or outperform existing normalization methods [28]. It removes unwanted variation by regressing out variability explained by the control probes present on the array. Finally, we use surrogate variable analysis (SVA) [29] for batch effect correction and adjustment for latent variables, a choice validated by the findings of our benchmarking [30]. SVA also increases statistical power by removing (unwanted) variability through aggregating information at the data level and constraining the data’s variability to the phenotype of interest [31]. Of 262 samples initially loaded onto the DNA methylation array, five data files were missing and one AF-alb result was missing. After visual inspection of density plots, one sample was removed for displaying an abnormal distribution. In addition, six samples were replicates and six samples were found to be mismatched for sex. This left 243 samples for further analysis.

### 3.6. Assessment of Cell Mixture Distribution

Quantile-normalized data were used to predict WBC subtypes based on a peripheral blood reference panel [32] using the minfi package [26]. Proportions of six cell types (B, CD4+ T, CD8+ T, granulocytes, monocytes and NK) were determined. SVA was also used as a reference-free method to adjust for differences in WBC composition [33].

### 3.7. Statistical Analysis, Dimension Reduction and Region-Level Methylation Analysis

Methylation beta-values (i.e., the ratio of the methylated probe intensity and the overall intensity), ranging from 0 to 1, were used for data plotting and (biological) interpretation. A zero-centered logarithmic transformation (M-value, calculated as the log2 ratio of the intensities of methylated probe vs. unmethylated probe) was used for most statistical analyses since they are more statistically valid, particularly in differential methylation analysis [34]. M-values were interrogated for association with Af-alb levels by modelling the study variables and potential confounders together with latent surrogate variables in a robust linear regression, using the R software. The distribution of Af-alb values at 6, 12 and 18 months was not Gaussian (*p*-value < 2.2 × 10^−16^ for all 3 times points) and we performed a logarithmic transformation obtaining data conforming more closely to the normal distribution (*p*-value = 0.07 and 0.002 for time point 12 and 18 months respectively) (Figure S1A). Statistical models for association of AF-alb with DNA methylation were adjusted (methylation = AF-alb levels + child sex + WBC types + Sample_Plate + Ethnicity + Season of sample collection), wherein Sample_Plate represents the HM450 batch of 96 samples each (Figure S1C). A dimension reduction approach (DMRcate) was implemented to reduce the ~450,000 individual sites into clusters of genetically proximal and correlated CpGs to enhance statistical power [31] and aid biological inferences (as single CpG sites often have more subtle functional relevance than CpG clusters), as per our previous work [35,36]. We used the recommended DMRcate proximity-based criteria and a minimum of two methylation sites per region with a maximum gap of 500 bp. To reduce the number of false positive results, we controlled for multiple testing using Benjamini Hochberg (FDR) procedure [37]. Identified differentially methylated regions (DMRs) were are reported if their FDR-adjusted *p*-values were < 0.05.

Two approaches to analysis of data were taken (Supplementary files S1C,D). Firstly, the continuous model assessed the association between DNA methylation and AF-alb levels from 18 months (at which the highest levels of exposure were observed and which is closest in time to the sampling of white blood cells for methylation analysis). Secondly, the levels of AF-alb were categorised depending on time and dose, specifically based on results for measurements at 6, 12 and 18 months, on whether levels were above or below the median, and whether this changed between time points. This categorical time-dose model consists of three categories: “low”, where AF-alb was low at each timepoint; “recurrent”, where AF-alb was high during the last two time points (12 and 18 months); and “intermittent”, where AF-alb was low or high at different time points (Supplementary files S1D).

## 4. Discussion

In this manuscript, we report changes in DNA methylation associated with aflatoxin exposure in WBC of Gambian children who are naturally exposed to aflatoxin in their diet. AF-alb is an established biomarker of aflatoxin exposure that reflects dietary intake of aflatoxin [12,38]. Chronic aflatoxin exposure in this population is known to be high [39], largely due to the inclusion of home-farmed peanuts as a dietary staple, and the impact of the exposure on child growth and liver disease has been reported in this population and others [18,40,41,42]. We have previously shown that exposure to aflatoxin in utero, as assessed by measurement of maternal AF-alb during pregnancy, is associated with differential DNA methylation in the WBC of Gambian infants at 6 months old [20]. Here, we have observed changes in DNA methylation in WBC of infants from the same population (but not the same children) at 2 years old, associated with levels of AF-alb during early life. The changes in DNA methylation were observed across a number of genes and pathways, with associations reported according to two methods of modelling the aflatoxin exposure- using a continuous model of AF-alb at 18 months (six months prior to blood sampling for epigenetic analysis) and a categorical model that reflects both time and dose response for aflatoxin exposure from six to eighteen months. In each case, analyses revealed DMPs and DMRs associated with AF-alb. Reassuringly, there is a notable amount of overlap between differentially methylated sites and regions across the analytical models.

Most of the associations observed were for small differences in DNA methylation, so care must be taken in attributing biological significance to the findings. Nevertheless, it is of interest to consider the genes and pathways in which differential DNA methylation was observed. In the model using AF-alb as a continuous variable, seven DMPs were observed. Four of these genes code for enzymes (SPR, an aldo-keto reductase; SESN1, a member of the sestrin family that play a role in the cellular response to oxidative stress; ME3, malic enzyme, which catalyzes the oxidative decarboxylation of malate to pyruvate using either NAD+ or NADP+ as a cofactor; CAPN5, a calcium-dependent cysteine protease involved in signal transduction in a variety of cellular processes), of which three are involved in response to oxidative stress. The others are BIRC8, an IAP-like protein 2 that is involved in pathways in apoptosis signalling and TNF signalling pathways; CBLN2, which may play a role in induction of synaptogenesis; and MAP7, a microtubule associated protein predominantly expressed in cells of epithelial origin and thought to be involved in microtubule dynamics.

A large number of DMRs were obtained (509), of which a large proportion encompassed at least three CpG sites, strengthening the reliability of these results. It is of interest that many DMRs were enriched in genes associated with hepatic failure, as the liver is the main target organ for the effects of aflatoxin, including acute poisoning and liver cancer [43]. This also highlights the ability of epigenetic markers to capture the biological mechanisms by which aflatoxin exposure leads to liver disease. Aflatoxin has also been associated with child growth impairment in several studies [18,38] and immune system effects [44]. In our data, aflatoxin-associated DMRs were enriched in inflammatory pathways and signalling events affecting the growth hormone-insulin-obesity axis, as well as Wnt signalling (involved in embryogenesis). Some of the DMRs relate to developmental disorders associated with reduced growth, such as brachydactyl syndrome, short neck and intrauterine growth retardation. Indeed, aflatoxin association in utero has been associated with impaired birthweight and growth in the infant [45]. Other DMRs observed from our data are in pathways involved in nervous system signalling events, blood clotting and morphogenesis.

The categorical time-dose model was used to explore whether changes in recurrence of exposure level over time affected the DNA methylation patterns. The children in Gambia are largely exclusively breast fed until five to six months of age [46]. During this period there may be some exposure to aflatoxin in the form of the metabolite aflatoxin M1 excreted in breast milk, which is an oxidised, and much less toxic metabolite of aflatoxin B1 [47]. Consequently, AF-alb levels tend to be very low in infants at 6 months, increasing with age as the children eat more family food [48]. This pattern is reflected in our data. However, as aflatoxin contamination of food is heterogeneous and will vary from family to family over time, and by season, levels of AF-alb in blood may be high in children at one sampling point and low at another, or vice versa. This complicates the interpretation of the role of the exposure in modifying DNA methylation, and it is not clear which time points may have a greater effect. The categorical time-dose model, based on the categorical split of AF-alb levels into high vs. low (based on the median value) attempts to take this variation into consideration. A small number of these DMPs showed sex related differences, but in general the results for boys and girls were similar in the continuous and categorical time-dose models.

The categorical time-dose model for recurrent vs. low AF-alb resulted in over 700 DMRs, of which 51 involved more than one CpG site with an effect size of more than 5%. Ontology analysis showed that DMRs were enriched in genes associated with cardiac and circulatory development. These genes were characterised by hypermethylation, whereas DMRs in genes from inflammatory and nervous system genes were mainly hypomethylated. When comparing the results between the models (Figure 5), the fact that the recurrent vs. low model (with the highest difference in AF-alb) yields a larger number of CpGs (FDR < 0.05) than the Recurrent vs. Intermittent or the Intermittent vs. Low models, is indicative of a time-dose response, which is further supported by a time-dose effect size observed for many of the yielded DMRs.

In our previous paper exploring epigenetic changes in relation to aflatoxin exposure in The Gambia, we compared DNA methylation in WBC of infants with AF-alb levels in serum of the mothers during pregnancy. We found differential DNA methylation in 71 DMPs associated with levels of maternal aflatoxin exposure. Of these, a small number were also found in this study. *PRKAR1B*, coding for a regulatory subunit of cAMP-dependent protein kinase A, was among genes in the DMRs for the Recurrent vs. Low AF-alb model and was also a DMP in this model. Mutations in the *PRKAR1B* gene cause neurodegenerative disorder [49]. Other genes that were DMP hits in the earlier paper that were found from one of the analytical models in the current study were *TGFB1* (encodes a secreted protein involved in cell growth and proliferation), *HAND2* (encodes a transcription factor essential for cardiac morphogenesis), *ZBTB16* (encodes a zinc finger transcription factor involved in cell cycle progression), *TNXB* (encodes a extracellular matrix glycoprotein), *AGAP3* (encodes an essential component of the NMDA receptor signalling complex) and *DNAJB6* (encodes a molecular chaperone involved in protein folding and assembly). Although only 10% of the genes from the earlier paper were hits in this study, it is interesting that these genes have shown differential DNA methylation in both 6-month-old children associated with aflatoxin exposure in pregnant mothers and in 24-month-old children, associated with aflatoxin exposure during early life.

A number of previous studies have reported changes in DNA methylation in relation to AFB1 treatment of human cells in vitro [50,51,52,53]. When primary human hepatocytes were treated with AFB1, Rieswijk et al. found six genes that showed persistent hypomethylation associated with up-regulated gene expression. One of these genes, *TXNRD1* (encodes an oxidoreductase) was a DMP in the recurrent vs. low AF-alb model. Wang et al. reported that increased methylation of the *RUNX3* gene was integral to the transformation of AFB_1_ treated LO2R cells. Methylation of a DMR including this gene was also seen to be associated with AF-alb in our Recurrent vs. Low AF-alb model. Modulation of the expression of the DNA methyl transferases, DNMT1 and DNTM3a, is a possible mechanism by which AFB1 may influence DNA methylation, leading to altered gene expression [54].

A limitation of the current study is that data on aflatoxin exposure of these infants in utero was not available. Our previous study on 6-month-old infants from the same Gambian population showed that maternal exposure to aflatoxin is associated with differential DNA methylation in the children, so it is possible that maternal exposure patterns may have contributed to DNA methylation in these children as well. Exposures at particular time points, such as in utero, may have a larger effect on DNA methylation changes induced by AFB1 than at other times. Nevertheless, it is interesting that 10% of genes with DMPs identified in the earlier study also showed up as affected in this dataset. We have taken into account several potential confounding factors in our analytical models. However, there will be other variables that could have impacted on DNA methylation of which we were not aware, or for which we do not have data. This is one of the reasons why we additional corrected for potential unknown effects using SVA (see Methods). Despite this, there is good evidence that aflatoxin B_1_ exposure in early life does modulate DNA methylation, and with several genes showing up in previous studies in vitro and in vivo.

The high levels of aflatoxin exposure in children in Gambia reported here and in other populations in low- and middle-income countries, highlight the need for aflatoxin reduction strategies, which have been discussed elsewhere [55].

In summary, our results reveal that dietary exposure to aflatoxin B_1_ during the first two years of life is associated with differential DNA methylation in a range of genes and pathways, some of which directly relate to known health effects of aflatoxin exposure. It is not clear whether the differences in DNA methylation associated with aflatoxin exposure are biologically significant. There was an apparent time-dose relationship in response and overlap of hits between analytical models. This provides further insight into potential mechanisms underlying the impact of exposure to aflatoxin on human health in early life periods. Further research into the role of DNA methylation on health effects of aflatoxin is warranted.

## Figures and Tables

**Table 1:** Characteristics of children in current study and AF-alb levels.

Variable	*n* (%)
Total	243 (100)
Male	127 (52)
Female	116 (48)
Ethnicity	
Fula	16 (7)
Jola	5 (2)
Mandinka	222 (91)
Birth weight (kg)	**Mean ± SD**
	3.03 ± 0.38
AF-alb (pg/mg)	**GM (95% CI)**
6 months (*n* = 230)	3.78 (3.29, 4.34)
12 months (*n* = 220)	25.1 (21.67, 29.13)
18 months (*n* = 210)	49.48 (43.34, 56.49)

## Data Availability

Data is available on request to A.G. (GhantousA@iarc.fr).

## Supplementary Materials

The following are available online at https://www.mdpi.com/article/10.3390/ijms22168967/s1.

## References

[1] Martin E.M., Fry R.C. (2018). Environmental Influences on the Epigenome: Exposure- Associated DNA Methylation in Human Populations. Annu. Rev. Public Health.

[2] Chung F.F.L., Herceg Z. (2020). The promises and challenges of toxico-epigenomics: Environmental chemicals and their impacts on the epigenome. Environ. Health Perspect..

[3] Zhang W., Song M., Qu J., Liu G.-H. (2018). Epigenetic Modifications in Cardiovascular Aging and Diseases. Circ. Res..

[4] Greenberg M.V.C., Bourc’his D. (2019). The diverse roles of DNA methylation in mammalian development and disease. Nat. Rev. Mol. Cell Biol..

[5] Jones P.A., Issa J.-P., Baylin P.A.J.S. (2016). Targeting the cancer epigenome for therapy. Nat. Rev. Genet..

[6] Bouras E., Karakioulaki M., Bougioukas K.I., Aivaliotis M., Tzimagiorgis G., Chourdakis M. (2019). Gene promoter methylation and can-cer: An umbrella review. Gene.

[7] Ling C. (2020). Epigenetic regulation of insulin action and secretion—Role in the pathogenesis of type 2 diabetes. J. Intern. Med..

[8] Stanzione R., Cotugno M., Bianchi F., Marchitti S., Forte M., Volpe M., Rubattu S. (2020). Pathogenesis of ischemic stroke: Role of epi-genetic mechanisms. Genes.

[9] Wadhwa P.D., Buss C., Entringer S., Swanson J.M. (2009). Developmental Origins of Health and Disease: Brief History of the Approach and Current Focus on Epigenetic Mechanisms. Semin. Reprod. Med..

[10] Küpers L., Monnereau C., Sharp G., Yousefi P., Salas L., Ghantous A., Page C., Reese R., Willcox A., Czamara D. (2019). Meta-analysis of epigenome-wide association studies in neonates reveals widespread differential DNA methylation associated with birthweight. Nat. Commun..

[11] IARC (2002). Some Traditional Herbal Medicines, Some Mycotoxins, Naphthalene and Styrene.

[12] Routledge M.N., Kimanya M.E., Shirima C.P., Wild C.P., Gong Y.Y. (2014). Quantitative correlation of aflatoxin biomarker with dietary intake of aflatoxin in Tanzanian children. Biomarkers.

[13] Shirima C.P., Kimanya M.E., Routledge M.N., Srey C., Kinabo J.L., Humpf H.-U., Wild C.P., Tu Y.-K., Gong Y.Y. (2015). A Prospective Study of Growth and Biomarkers of Exposure to Aflatoxin and Fumonisin during Early Childhood in Tanzania. Environ. Health Perspect..

[14] Watson S., Gong Y.Y., Routledge M.N. (2017). Interventions Targeting Child Undernutrition in Developing Countries May Be Undermined by Dietary Exposure to Aflatoxin. Crit. Rev. Food Sci. Nutr..

[15] Xu Y., Gong Y., Routledge M. (2018). Aflatoxin exposure assessed by aflatoxin albumin adduct biomarker in populations from six African countries. World Mycotoxin J..

[16] Kamala A., Shirima C., Jani B., Bakari M., Sillo H., Rusibamayila N., De Saeger S., Kimanya M., Gong Y., Simba A. (2018). Outbreak of an acute aflatoxicosis in Tanzania during 2016. World Mycotoxin J..

[17] Gong Y.Y., Watson S., Routledge M.N. (2016). Aflatoxin Exposure and Associated Human Health Effects, a Review of Epidemiological Studies. Food Saf..

[18] Watson S., Moore S.E., Darboe M.K., Chen G., Tu Y.-K., Huang Y.-T., Eriksen K.G., Bernstein R.M., Prentice A.M., Wild C.P. (2018). Impaired growth in rural Gambian infants exposed to aflatoxin: A prospective cohort study. BMC Public Health.

[19] Aguilar F., Hussain S.P., Cerutti P. (1993). Aflatoxin B1 induces the transversion of G-T in codon 249 of the P53 tumour suppressor gene in human hepatocytes. Proc. Natl. Acad. Sci. USA.

[20] Hernandez-Vargas H., Castelino J., Silver M.J., Dominguez-Salas P., Cros M.-P., Durand G., Le Calvez-Kelm F., Prentice A.M., Wild C.P., Moore S.E. (2015). Exposure to aflatoxin B1 in utero is associated with DNA meth-ylation in white blood cells of infants in The Gambia. Int. J. Epidemiol..

[21] Moore S.E., Fulford A.J., Darboe M.K., Jobarteh M.L., Jarjou L.M., Prentice A.M. (2012). A randomized trial to investigate the effects of pre-natal and infant nutritional supplementation on infant immune development in rural Gambia: The ENID trial: Early Nutrition and Immune Development. BMC Pregnancy Childbirth.

[22] Chapot B., Wild C.P. (1991). ELISA for quantification of aflatoxin-albumin adducts and their application to human exposure as-sessment. Tech. Diagn. Pathol..

[23] Winkler J., Xu Y., Gong Y.Y., Lindahl J., Kersten S., Dänicke S., Routledge M.N. (2019). Preliminary study on the relationship between af-latoxin-bovine serum albumin adducts in blood and aflatoxin M1 levels in milk of dairy cows. Mycotoxin Res..

[24] McCoy L.F., Scholl P.F., Sutcliffe A.E., Kieszak S.M., Powers C.D., Rogers H.S., Gong Y.Y., Groopman J.D., Wild C.P., Schleicher R.L. (2008). Hu-man aflatoxin albumin adducts quantitatively compared by ELISA, HPLC with fluourescence detection, and HPLC with isotope dilution mass spectrometry. Cancer Epidemiol. Prev. Biomark..

[25] Bibikova M., Barnes B., Tsan C., Ho V., Klotzle B., Le J.M., Delano D., Zhang L., Schroth G.P., Gunderson K.L. (2011). High density DNAmethylation rray with single CpG site resolution. Genomics.

[26] Aryee M.J., Jaffe A.E., Corrada-Bravo H., Ladd-Acosta C., Feinberg A.P., Hansen K.D., Irizarry R.A. (2014). Minfi: A flexible and comprehen-sive Bioconductor package for the analysis of Infinium DNA methylation microarrays. Bioinformatics.

[27] Chen Y.-A., Lemire M., Choufani S., Butcher D.T., Grafodatskaya D., Zanke B.W., Gallinger S., Hudson T.J., Weksberg R. (2013). Discovery of cross-reactive probes and polymorphic CpGs in the Illumina Infinium HumanMethylation450 microarray. Epigenetics.

[28] Fortin J.-P., Labbe A., Lemire M., Zanke B.W., Hudson T.J., Fertig E.J., Greenwood C.M., Hansen K.D. (2014). Functional normalization of 450 k methylation array data improves replication in large cancer studies. Genome Biol..

[29] Parker H.S., Bravo H.C., Leek J.T. (2014). Removing batch effects for prediction problems with frozen surrogate variable analysis. PeerJ.

[30] Perrier F., Novaloaca A., Ambatipudi S., Baglietto L., Ghantous A., Perduca V., Barrdahl M., Harlid S., Ong K.K., Cardona A. (2018). Identi-Fying and correcting epigenetics measurements for systematic sources of variation. Clin. Epigenetics.

[31] Lin X.C., Barton S., Holbrook J.D. (2016). How to make DNA methylome wide association studies more powerful. Epigenomics.

[32] Reinius L.E., Acevedo N., Joerink M., Pershagen G., Dahlén S.E., Greco D., Söderhäll C., Scheynius A., Kere J. (2012). Differential DNA methylation in purified human blood cells: Implications for cell lineage and studies on disease susceptibility. PLoS ONE.

[33] Kaushal A., Zhang H., Karmaus W.J.J., Ray M., Torres M.A., Smith A.K., Wang S.-L. (2017). Comparison of different cell type correction methods for genome-scale epigenetics studies. BMC Bioinform..

[34] Du P., Zhang X.A., Huang C.C., Jafari N., Kibbe W.A., Hou L.F., Lin S.M. (2010). Comparison of Beta-value and M-value methods for quan-tifying methylation levels by microarray analysis. BMC Bioinormatics.

[35] Ambatipudi S., Cuenin C., Hernandez-Vargas H., Ghantous A., Le Calvez-Kelm F., Kaaks R., Barrdahl M., Boeing H., Aleksan-drova K., Trichopoulou A. (2016). Tobacco smoking-associated ge-nome-wide DNA methylation changes in the EPIC study. Epigenomics.

[36] Woo H.D., Fernandez-Jimenez N., Ghantous A., degli Esposti D., Cuenin C., Cahais V., Choi I.J., Kim Y.-I., Kim J., Herceg Z. (2018). Genome-wide profiling of normal gastric mucosa identifiesHelicobacter pylori- and cancer-associated DNA methylome changes. Int. J. Cancer.

[37] Benjamini Y., Hochberg Y. (1995). Controlling the False Discovery Rate: A Practical and Powerful Approach to Multiple Testing. J. R. Stat. Soc. Ser. B.

[38] Wild C.P., Hudson G.J., Sabbioni G., Chapot B., Hall A.J., Wogan G.N., Whittle H., Montesano R., Groopman J.D. (1992). Dietary intake of aflatoxins and the level of albumin-bound aflatoxin in peripheral blood in The Gambia, West Africa. Cancer Epidemiol. Prev. Biomark..

[39] Castelino J.M., Dominguez-Salas P., Routledge M.N., Prentice A.M., Moore S.E., Hennig B.J., Wild C.P., Gong Y.Y. (2014). Seasonal and gesta-tion stage associated differences in aflatoxin exposure in pregnant Gambian women. Trop. Med. Int. Health.

[40] Gong Y.Y., Hounsa A., Egal S., Turner P.C., Sutcliffe A.E., Hall A.J., Cardwell K., Wild C.P. (2004). Postweaning Exposure to Aflatoxin Results in Impaired Child Growth: A Longitudinal Study in Benin, West Africa. Environ. Health Perspect..

[41] Gong Y.Y., Wilson S., Mwatha J.K., Routledge M.N., Castelino J.M., Zhao B., Kimani G., Kariuki H.C., Vennervald B., Dunne D. (2012). Aflatoxin Exposure May Contribute to Chronic Hepatomegaly in Kenyan School Children. Environ. Health Perspect..

[42] Castelino J.M., Routledge M.N., Wilson S., Dunne D.W., Mwatha J.K., Gachuhi K., Wild C.P., Gong Y.Y. (2015). Aflatoxin exposure is inversely associated with IGF1 and IGFBP3 levels in vitro and in Kenyan schoolchildren. Mol. Nutr. Food Res..

[43] Schrenk D., Bignami M., Bodin L., Chipman J.K., del Mazo J., Grasl-Kraupp B., Hogstrand C., Hoogenboom L., Leblanc J.C., EFSA Panel on Contaminants in the Food Chain (CONTAM) (2020). Risk assessment of aflatoxins in food. EFSA J..

[44] Turner P.C., Moore S.E., Hall A.J., Prentice A.M., Wild C.P. (2003). Modification of immune function through exposure to dietary aflatoxin in Gambian children. Environ. Health Perspect..

[45] Smith L.E., Prendergast A.J., Turner P.C., Humphrey J.H., Stoltzfus R.J. (2017). Aflatoxin exposure during pregnancy, maternal anemia, and adverse birth outcomes. Am. J. Trop. Med. Hyg..

[46] Eriksen K.G., Johnson W., Sonko B., Prentice A.M., Darboe M.K., Moore S.E. (2017). Following the World health Organization’s recom-mendation of exclusive breastfeeding to 6 months of age does not impact the growth of rural Gambian infants. J. Nutr..

[47] Zarba A., Wild C.P., Hudson G.J., Hall A.J., Montesano R., Groopman J.D. (1992). Aflatoxin M1 in human breast milk from The Gambia, West Africa, quantified by combined monoclonal antibody immunoaffinity chromatography and HPLC. Carcinogenesis.

[48] Gong Y.Y., Egal S., Hounsa A., Turner P.C., Hall A.J., Cardwell K.F., Wild C.P. (2003). Determinants of aflatoxin exposure in young children from Benin and Togo, West Africa: The critical role of weaning. Int. J. Epidemiol..

[49] Wong T.H., Chiu W.Z., Breedveld G.J., Li K.W., Verkerk A.J.M.H., Hondius D., Hukema R., Seelaar H., Frick P., Severijnen L.-A. (2014). PRKAR1B mutation associated with a new neurodegenerative disorder with unique pathology. Brain.

[50] Rieswijk L., Claessen S.M.H., Bekers O., van Herwijnen M., Theunissen D.H.J., Jennen D.G.J., de Kok T.M.C.M., Kleinjans J.C.S., van Breda S.G.J. (2016). Aflatoxin B1 induces persistent epigenomic effects in primary human hepatocytes associated with hepatocellular car-cinoma. Toxicology.

[51] Wang S., He Z., Li D., Zhang B., Li M., Li W., Zhu W., Xing X., Zeng X., Dong G. (2017). Aberrant methylation of RUNX3 is present in aflatoxin B1-induced transformation of the LO2R cell line. Toxicology.

[52] Soni P., Ghufran M.S., Kanade S.R. (2018). Aflatoxin B1 induced multiple epigenetic modulators in human epithelial cell lines. Toxicon.

[53] Trydyak V., Borowa-Mazgay B., Beland F.A., Pogribny I.P. (2019). Gene expression and cytosine DNA methylation alterations in in-duced pluripotent stem cell-derived human hepatocytes treated with low doses of chemical carcinogens. Arch. Toxicol..

[54] Zhou X., Gan F., Hou L., Liu Z., Su J., Lin Z., Le G., Huang K. (2019). Aflatoxin B1 induces immunotoxicity through the DNA methyltrans-ferase-mediated JAK2/STAT3 pathway in 3D4/21 cells. J. Agric. Food Chem..

[55] Ismail A., Naeem I., Gong Y.Y., Routledge M.N., Akhtar S., Riaz M., Ramalho L.N.Z., de Oliveira C.A.F., Ismail Z. (2021). Early life exposure to dietary aflatoxins, health impact and control perspectives: A review. Trends Food Sci. Technol..

