# Supplementary material for: Aflatoxin Exposure during Early Life Is Associated with Differential DNA Methylation in Two-Year-Old Gambian Children
Source: Int J Mol Sci. 2021 Aug 20;22(16):8967. doi: 10.3390/ijms22168967 (PMC8396526; doi:10.3390/ijms22168967)
Supplement: Supplementary file 1 [file ijms-22-08967-s001.zip › Ghantous et al supplementary files/Ghantous et al_Supplementary file S5.pptx]

## Slide 1
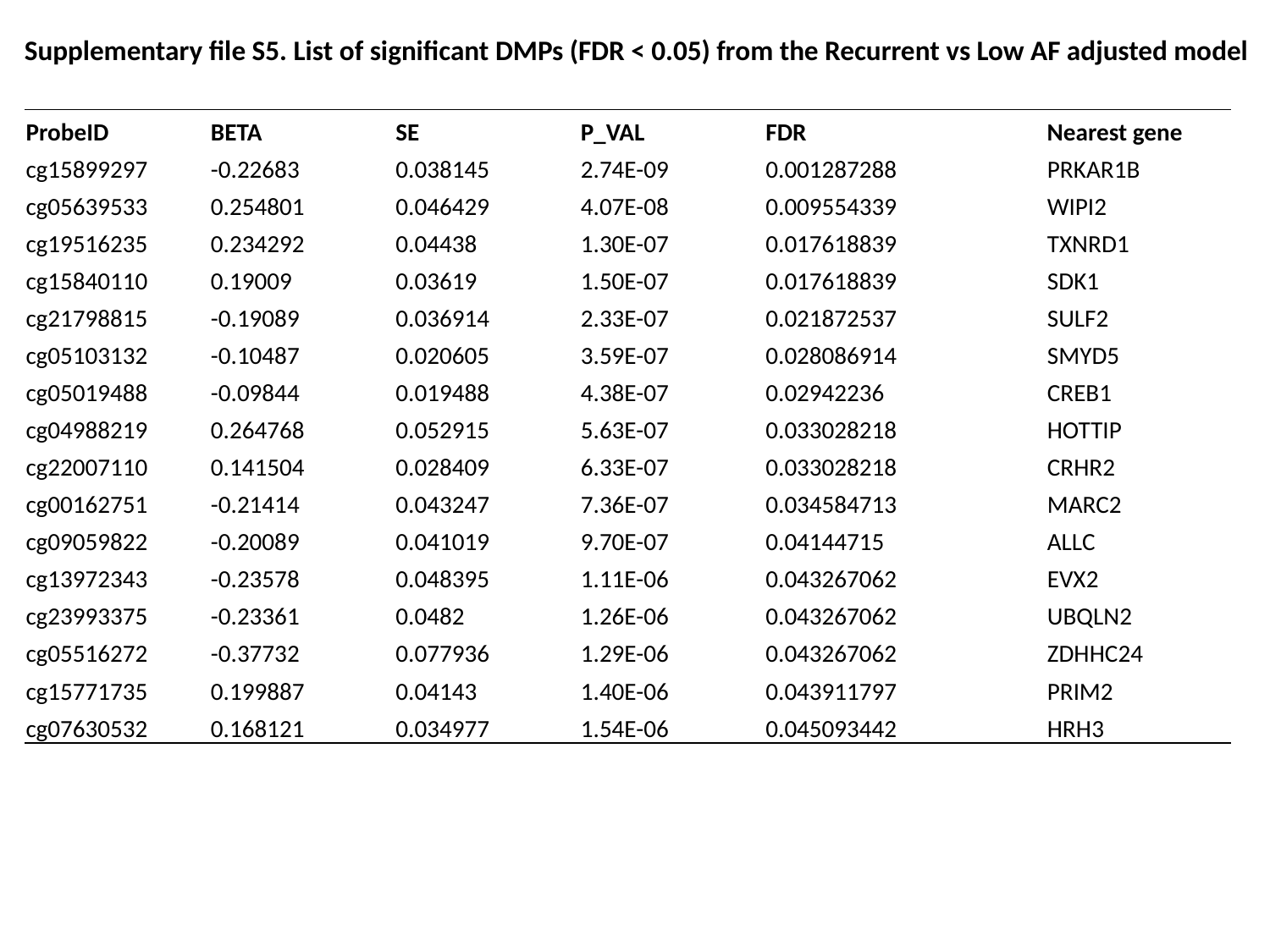

Supplementary file S5. List of significant DMPs (FDR < 0.05) from the Recurrent vs Low AF adjusted model
| ProbeID | BETA | SE | P\_VAL | FDR | Nearest gene |
| --- | --- | --- | --- | --- | --- |
| cg15899297 | -0.22683 | 0.038145 | 2.74E-09 | 0.001287288 | PRKAR1B |
| cg05639533 | 0.254801 | 0.046429 | 4.07E-08 | 0.009554339 | WIPI2 |
| cg19516235 | 0.234292 | 0.04438 | 1.30E-07 | 0.017618839 | TXNRD1 |
| cg15840110 | 0.19009 | 0.03619 | 1.50E-07 | 0.017618839 | SDK1 |
| cg21798815 | -0.19089 | 0.036914 | 2.33E-07 | 0.021872537 | SULF2 |
| cg05103132 | -0.10487 | 0.020605 | 3.59E-07 | 0.028086914 | SMYD5 |
| cg05019488 | -0.09844 | 0.019488 | 4.38E-07 | 0.02942236 | CREB1 |
| cg04988219 | 0.264768 | 0.052915 | 5.63E-07 | 0.033028218 | HOTTIP |
| cg22007110 | 0.141504 | 0.028409 | 6.33E-07 | 0.033028218 | CRHR2 |
| cg00162751 | -0.21414 | 0.043247 | 7.36E-07 | 0.034584713 | MARC2 |
| cg09059822 | -0.20089 | 0.041019 | 9.70E-07 | 0.04144715 | ALLC |
| cg13972343 | -0.23578 | 0.048395 | 1.11E-06 | 0.043267062 | EVX2 |
| cg23993375 | -0.23361 | 0.0482 | 1.26E-06 | 0.043267062 | UBQLN2 |
| cg05516272 | -0.37732 | 0.077936 | 1.29E-06 | 0.043267062 | ZDHHC24 |
| cg15771735 | 0.199887 | 0.04143 | 1.40E-06 | 0.043911797 | PRIM2 |
| cg07630532 | 0.168121 | 0.034977 | 1.54E-06 | 0.045093442 | HRH3 |
