# Supplementary material for: Aflatoxin Exposure during Early Life Is Associated with Differential DNA Methylation in Two-Year-Old Gambian Children
Source: Int J Mol Sci. 2021 Aug 20;22(16):8967. doi: 10.3390/ijms22168967 (PMC8396526; doi:10.3390/ijms22168967)
Supplement: Supplementary file 1 [file ijms-22-08967-s001.zip › Ghantous et al supplementary files/Ghantous et al_Supplementary file S6.pptx]

## Slide 1
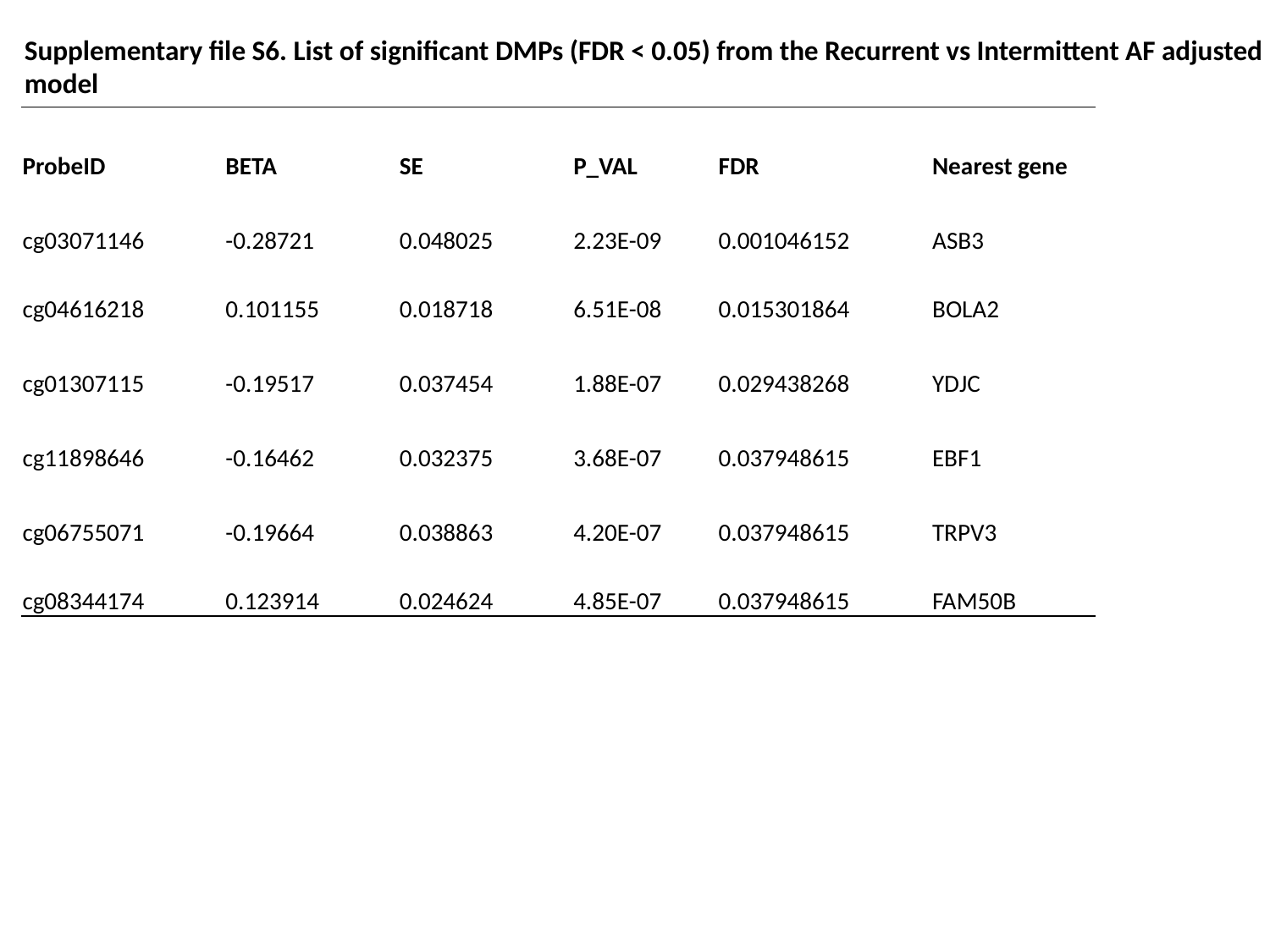

Supplementary file S6. List of significant DMPs (FDR < 0.05) from the Recurrent vs Intermittent AF adjusted model
| ProbeID | BETA | SE | P\_VAL | FDR | Nearest gene |
| --- | --- | --- | --- | --- | --- |
| cg03071146 | -0.28721 | 0.048025 | 2.23E-09 | 0.001046152 | ASB3 |
| cg04616218 | 0.101155 | 0.018718 | 6.51E-08 | 0.015301864 | BOLA2 |
| cg01307115 | -0.19517 | 0.037454 | 1.88E-07 | 0.029438268 | YDJC |
| cg11898646 | -0.16462 | 0.032375 | 3.68E-07 | 0.037948615 | EBF1 |
| cg06755071 | -0.19664 | 0.038863 | 4.20E-07 | 0.037948615 | TRPV3 |
| cg08344174 | 0.123914 | 0.024624 | 4.85E-07 | 0.037948615 | FAM50B |
