# Supplementary figures and images for: Aflatoxin Exposure during Early Life Is Associated with Differential DNA Methylation in Two-Year-Old Gambian Children
Source: Int J Mol Sci. 2021 Aug 20;22(16):8967. doi: 10.3390/ijms22168967 (PMC8396526; doi:10.3390/ijms22168967)

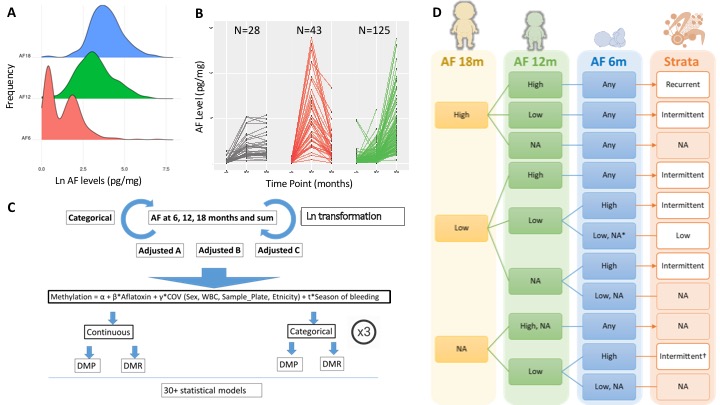

Supplement: Supplementary file 1 [file ijms-22-08967-s001.zip › Ghantous et al supplementary files/Ghantous et al_Supplementary file S1.jpg]

## Slide 1
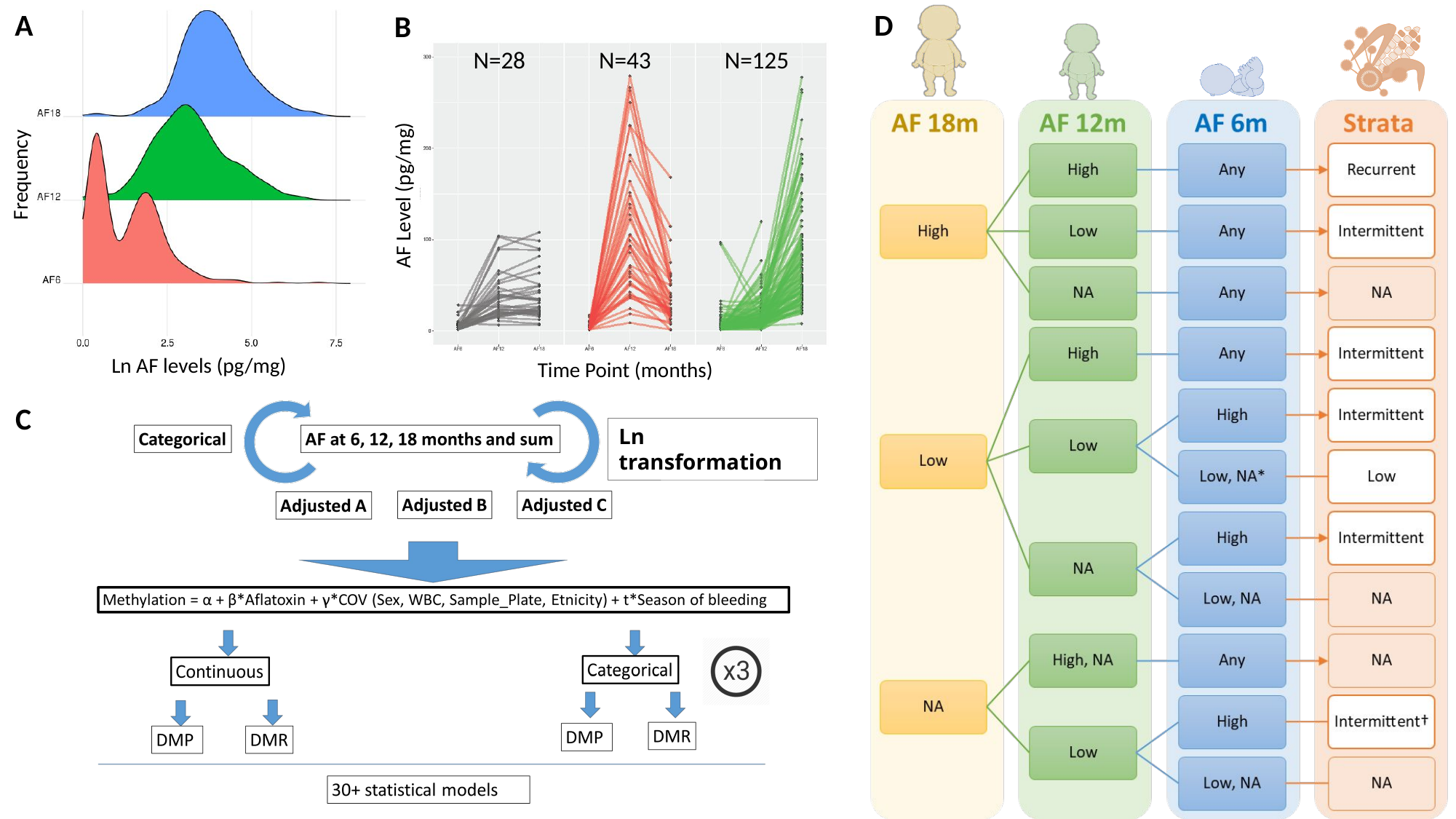

D
A
B
N=28
N=43
N=125
Frequency
AF Level (pg/mg)
Ln AF levels (pg/mg)
Time Point (months)
C
Ln transformation

Supplement: Supplementary file 1 [file ijms-22-08967-s001.zip › Ghantous et al supplementary files/Ghantous et al_Supplementary file S1.pptx]

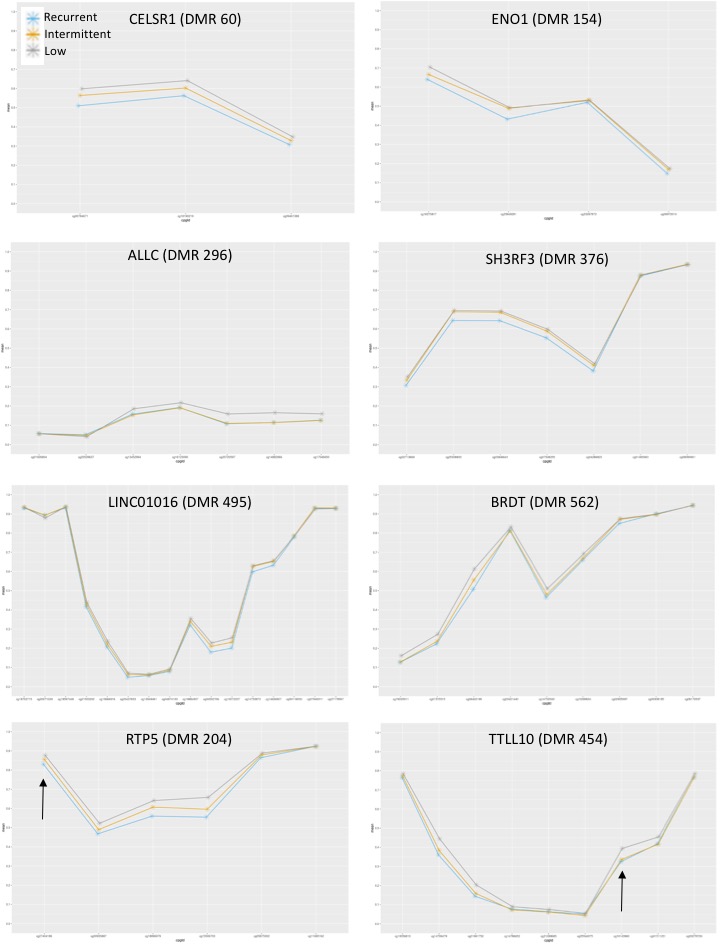

Supplement: Supplementary file 1 [file ijms-22-08967-s001.zip › Ghantous et al supplementary files/Ghantous et al_Supplementary file S9.jpg]
